# An Alternative Current Device to Simplify Leakage Detection in Complex DC Systems

**DOI:** 10.3390/s26092803

**Published:** 2026-04-30

**Authors:** Brunalice de Matos Mercer, Rodrigo Antonio Sbardeloto Kraemer, Luis Otavio Steffenmunsberg Grillo, Durval da Silva Neto, Henrique Monteiro Basso, Mauricio Ibarra Dobes, Marcos Damont Terra

**Affiliations:** 1 CERTI Foundation, Florianópolis 88040-970, SC, Brazil; brunamercer@gmail.com (B.d.M.M.); lrg@certi.org.br (L.O.S.G.); dsn@certi.org.br (D.d.S.N.); hem@certi.org.br (H.M.B.); mcd@certi.org.br (M.I.D.); 2 Engie Brasil Energia, Florianópolis 88025-255, SC, Brazil; marcos.terra@engie.com

**Keywords:** DC leakage current detection, ground fault localization, DC systems

## Abstract

An alternative, low-cost, current device to be used in leakage detection is presented in this work. The main advantages, besides the cost and portability, are the high efficiency and ease of operation, enabling a simplified and effective implementation in energized electrical power systems. Its main purpose includes detections in direct current auxiliary systems (DCAS), whose reliable and continuous operation is essential to guarantee safety and robustness in a large variety of assets, such as large power plants, substations and even industry. Such effectiveness along with the proof of concept are demonstrated through tests and real maintenance situations exhibited in the final sections.

## 1. Introduction

Protection in electrical power systems involves several steps from constant monitoring, collaborating with asset management, until the execution of local procedures to detect specific problems. In this sense, the detection and investigation of leakage current in electrical circuits, especially those affecting direct current auxiliary systems (DCAS), are indispensable and must be included in periodic maintenance of power plants, substations and other large size installations.

The leakage current may be defined as an unintended current flow through insulation failures, and when it occurs in DC auxiliary systems (DCAS), it can be highly damaging [1,2].

DCASs are essential for the proper operation of large power systems, as they supply energy for control, command, and protection functions. These systems typically support a wide range of critical equipment, including protection relays, control and monitoring devices, teleprotection and telemetering systems, circuit breaker actuation mechanisms, battery chargers, and emergency lighting.

From an operational perspective, the supplied loads can be classified according to their required level of supply continuity. Permanent loads, usually fed by DC, require uninterrupted operation and are directly associated with system protection and control. Essential loads, typically supplied by AC, can tolerate short interruptions and include equipment such as battery chargers and switching device actuators. Non-essential loads allow longer interruptions and are related to auxiliary services such as cooling, heating, and general infrastructure systems. Additionally, emergency loads are designed to ensure minimum operational visibility and safety during power loss conditions.

DCASs are usually designed as ungrounded (isolated) systems, meaning that their poles are not directly connected to ground. This configuration allows the system to continue operating even in the presence of a single fault, thereby increasing overall reliability and availability [3].

Although this grounding scheme is appropriate to guarantee the continuity of essential devices, in the occurrence of a second fault or interference, a short circuit may occur in the DCAS, damaging essential components and affecting the proper action of protection, disabling relays and breakers, and even causing trips in primary machines, like generators.

During a leakage current situation in the DCAS, two tasks are required: detecting the fault and locating it. Detecting a fault to ground is simple and several methods can be used, such as indicator lights, voltmeters, relays, specific monitoring devices, etc. However, most of these detection methods are not selective, meaning that they can detect but not find the fault. To actually find the fault, the most common approaches include selective disconnection of circuits, aiming at fault isolation, and the tracking method using a signal injector—which represents the device proposed here, and a portable detector [4].

This last approach has the main advantage of allowing work on the energized circuit, not disturbing the operation of the plant. In fact, for some power plants or substations, a shutdown to search for leakage current can cost a lot of money, besides risking some main parts of the installation.

It is important to highlight the leakage current may be very small and, even in the presence of a fault, may not be detected by some methods, so the relevance of a methodology that can insert a signal into the system that will flow through a possible failure.

Although Brazilian standard NBR 5410 [5], for low voltage electrical installations, does not demand or even recommend methodologies to find leakage, many devices that inject a current signal into the system have been commercialized by different manufacturers [6,7,8,9,10], with emphasis on this last device from Bender. By checking some similarities among them, regarding features and price, it can be inferred that most of them rely on the same principle, something that the proposed device attempts to differentiate itself.

One of the most well known devices is the Bender model, which has plenty of functionalities and will serve as a comparison set for the design presented here.

In this sense, this article proposes an alternative device that, unlike the more complex and expensive commercial devices investigated, is distinguished by its simplicity that allows its widespread implementation in the electricity sector, especially in solar plants, which have been growing exponentially in quantity every day.

The device presents some conceptual and structural advantages against its main competitors that, combined with its low cost and effectiveness in quickly resolving leakage problems, can significantly contribute to maintenance processes.

Designed with the intended for use in a large hydroelectric plant in Brazil, its adaptability allows its use in DCASs installed in different typologies. The proposed schematic consists of a signal injector that, unlike traditional models that are based on self-adaptive current (relying on the circuit impedance), works actively, enabling an active variation in its current according to the circuit’s needs, being suitable for various applications.

Furthermore, using it with a commercial clamp meter allows for a broad yet effective scan across the entire system, reducing the time to locate the faulty circuit and providing an accurate diagnosis, which is important especially considering the time and operational costs involved. The proposal and its proof of concept in an operational environment are demonstrated in this paper.

## 2. Leakage Current Detection in Auxiliary DC Systems

It is not unusual for electrical installations, some current might flow undesirably to ground through the protective ground conductor or remains at the surroundings insulation [2]. This current is usually referred to as leakage current, which occurs in abnormal or faulty conditions, and frequently flows in the insulation surrounding the conductor, which can reach the earth wire, accessible metal parts of enclosures and other meaningful parts of electrical systems [1].

In electrical power systems, the occurrence of leakage current may affect a vital part, the DCAS, which is a critical, low-voltage, DC system that supplies power for control, protection, and automation functions. These systems usually include batteries, rectifiers, battery chargers, distribution systems, switching and protective devices, and additional control and monitoring equipment [11].

Usually, the DCAS is designed with an isolated grounding scheme, which is ideal for ensuring the continuity of its systems (protection, control) in the occurrence of a fault, but whose operability might be compromised if a first fault/leakage occurs and is not promptly located and eliminated [12].

In Brazilian electrical installations, regulation demands that the NBR 5410 standard must be followed to guarantee safety and proper function of low voltage systems, such as the DCAS. This standard rules that such systems should be designed in a way that faults on its circuits must not compromise the proper behavior of controlled devices or even, its design should avoid automatic disconnection in the occurrence of a first fault. To provide such features, these auxiliary systems usually contain IMDs (insulation monitoring devices), which can successfully identify leakage but not its source or exact the circuits through which it flows.

For the NBR 5410 standard, there is no requirement for additional devices or procedures that should properly locate the leakage. But to be able to locate such occurrences in a fast and simple way can avoid serious hazards and losses (financial or physical) to the system.

### 2.1. Non-Measurement Risks

The inadequacies in the materials that make up the components, such as capacitors and semiconductors, are the main cause of leakage current [13]. Electrical systems often present high-resistance insulation, usually preventing current from leaking. However, if the insulation is old or damaged, the resistance is reduced and a small amount of current may flow. In addition, longer conductors, with greater inherent capacitance, may exhibit more leakage. Electronic equipment is another source of leakage, as their filter capacitors contribute to the overall level of leakage current [2].

Circuits that have protection equipment, such as ground fault current interrupters, may experience unnecessary and intermittent tripping due to leakage current. In extreme cases, it can also cause a voltage rise in accessible conductive parts, compromising installation safety.

As previously established, the DCAS is a vital part of an electrical power system and can be considered as one of the most critical components of a protection, control, and monitoring scheme. Usually, DCASs are typically non-redundant, requiring highly reliable operation. Failure to detect leakage or faults can lead to malfunctions, such as protection devices becoming unable to detect faults, breakers failing to trip, and loss of local or remote indications [11]. These occurrences can lead to serious damage in the operation of power plants or substations.

It is known that the lack of precision in identifying and locating leakage in DCASs has already caused interruptions in electrical power systems. Failures in some parts of the system can even trigger a sequence of events that may permanently damage assets of the plant and affect the energy supply to final users [3].

In fact, one of the main reasons that led to the development of the device presented here was a trip occurrence in one of the generating units of the Ponte da Pedra Hydroelectric Power Plant, requiring an extensive investigation regarding methods to find leakage current.

In the process of eliminating leakage current, it is important to quantify it current and identify the original source. Many devices, such as specific relays and IMDs, help in detecting leakage, but fail in locating the source to effectively eliminate the problem. Therefore, devices such as the one proposed in this work are essential in protecting the system [2].

### 2.2. Main Approaches for Leakage Detection

A critical point in maintenance is the search for insulation failures that lead to leakage current problems. There are fixed and portable devices available, and choosing the proper method can save time and reduce losses in installations. Unfortunately, what can be perceived, specifically in Brazil, is that non-conforming methods to detect and locate leakage are widely used by technical teams. These methods are usually inefficient and can cause even more disturbance to the system [12].

The most common methods to locate a leakage point are the fault isolation through a selective disconnection of the circuit, and the tracking method using an injected signal and a portable detector [3]. A third general alternative could use an additional independent DC power supply, allowing the open-circuit condition to be reproduced without causing outages.

In fact, network switching performed through selective disconnection is the simplest and most widely used method. However, due to the need to de-energize and test each feeder individually, the process can take long periods, often leading to postponements that can coincide with a scheduled production outage, bringing further risks to the system [14].

Additionally, some other alternative approaches that can help in leakage detection can include:

- The use of a megohmmeter, that can assist in testing the insulation;
- Frequency Domain Reflectometry (FDR), to measure sensitivity to cable insulations;
- Wheatstone bridge Methods, which can check for cable defects.

As previously mentioned, the signal injection methodology has been incorporated into several commercial devices, with most of them, like the one made by Bender, working on a self-adaptive current principle. Considering the main advantage of this approach, that enables working in an energized environment, the device proposed here gathers the same general principle for its applicability. Also, with the aid of a clamp meter working as a detector device, the equipment becomes fully operational to assess a variety of leakages.

Notably, some occurrences can even consider the isolated use of a current clamp meter, which is a common way to locate the leakage [2]. Nevertheless, this is a simplistic approach that demands more time and tests and might not be successful if the leakage is too small. Different from the signal generator proposed, if the leakage current is only a low insulation resistance failure without significant current, the clamp will not detect. On the other hand the signal induced in the system will expose such failure.

Therefore, using an adequate and portable device, like the one presented here—based on the signal injection method, can enable an early (and important) detection in insulation failures, avoiding unwanted interruptions in the operation of power plants and substations.

## 3. Proposal of an Alternative Device

A trip occurrence in one of the three main generators of a large hydropower plant in Brazil triggered a series of initiatives aimed to better understand the process of detecting leakage current and the most common techniques to locate it. It was verified that there is no pattern to such investigation and that one of the most known commercial equipment to detect leakage (Bender) could not solve that particular problem properly [15,16].

Ahead of this, the technical team decided to develop an alternative device that would overcome some drawbacks of the commercial option available at the power plant.

The developed device would be a current injector that would be assembled along with a specific commercial clamp to detect and locate the leakage. Its design, comprising an independent supply source and an adjustable method to inject current can bring meaningful improvements against other methods, especially due its capacity of injecting a variable controlled current into the circuit.

Furthermore, thanks to the use of simple components and techniques specifically developed for the leakage problem, the cost of the device remains much lower than current commercial alternatives, which is another strong attribute of the proposal.

### 3.1. Equivalent Circuit Model

The working principle of the device consists of generating an alternating test current (AC) limited to a level that minimizes the risk of interference with internal devices or unintended triggering of protection systems. From a design standpoint, this is achieved through AC coupling, galvanic isolation via transformer, and passive current limitation. As a result, the injected signal does not introduce a direct DC component into the system. Although transient responses during connection and disconnection were not captured using oscilloscope measurements, no abnormal behavior, such as protection relay misoperation or system disturbances, was observed during experimental validation in energized systems.

This pulse of current flows through the energized conductors until the point of leakage, the insulation failure. Once it reaches this point, the current returns to the device through the failure point and the grounding conductor, enabling the detection by an auxiliary device, which in this case will be a regular commercial current clamp.

In the absence of leakage (i.e., under high insulation resistance conditions), no closed current path is established, and therefore no measurable current is detected by the clamp meter. This condition is interpreted as normal system operation, indicating the absence of significant insulation faults.

**Figure 1:**
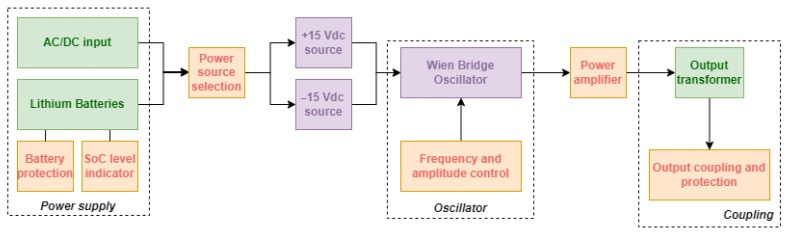
Block diagram for the proposed device.

The equivalent circuit comprises six main parts that include supply source connection, power source selection, DC voltage source, oscillator, amplifier and coupling, whose diagram is depicted in Figure 1. Their description is briefly detailed as follows.

**Power Supply Source Connection**. Includes two options for power supply: by grid connection, using a 100–220 Vac to 12 Vdc 3 A converter, or by lithium batteries. Internal battery power adds an additional feature to the device, allowing portability for use in remote locations.

**Power Source Selection**. It allows automatic switching to main power in situations where the power supply is supplied by the battery but a new connection is established. This input also has a protection scheme to prevent problems during reverse connection.

**Figure 2:**
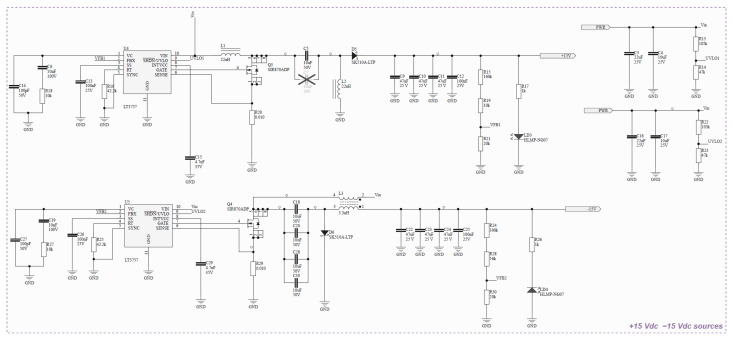
Schematic for DC voltage source ±15 Vdc.

**DC Voltage Source**. It provides symmetrical voltage according to the circuit’s needs. The power supply is built with two boost type switching sources, and the schematic is presented in Figure 2.

**Figure 3:**
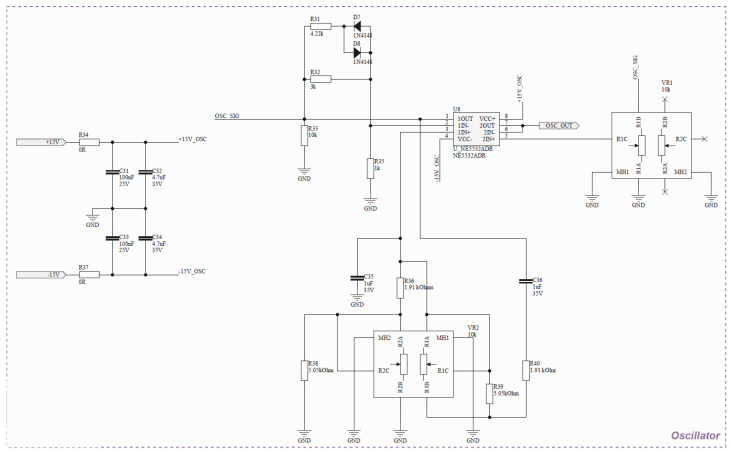
Schematic for oscillator: Wien b-ridge and the frequency and amplitude control.

**Oscillator**. This component is responsible for generating the test signal. It is implemented as a Wien bridge oscillator, which inherently produces a sinusoidal waveform. The output is AC-coupled through a capacitor to remove any DC offset and to symmetrically center the waveform around zero. It is important to note that no dedicated amplitude or temperature stabilization mechanism is employed, nor is there an active feedback loop to ensure long-term frequency or amplitude stability. As a result, the oscillator may exhibit some degree of non-linearity and drift, which are intrinsic characteristics of this topology when used without automatic gain control. Nevertheless, within the intended operating frequency range, the circuit maintains a predominantly sinusoidal waveform with acceptable distortion for the proposed application. The output voltage level is manually adjusted using potentiometers and verified through external measurement instruments, such as a multimeter or clamp meter, to ensure compliance with the required test conditions. The schematic is presented in Figure 3.

**Figure 4:**
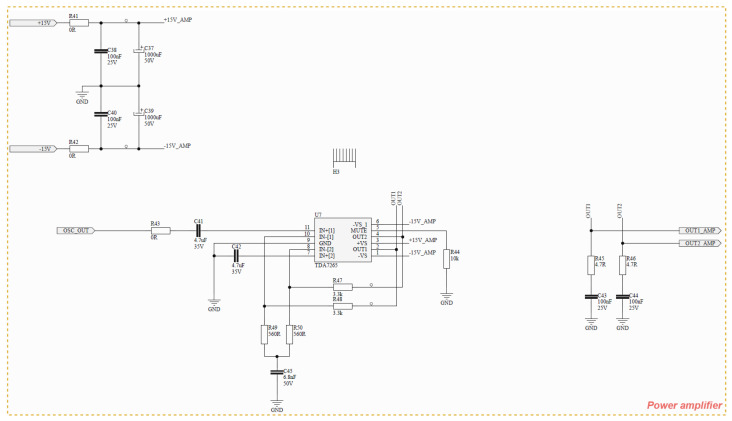
Schematic for power amplifier.

**Amplifier**. The amplifier stage increases the oscillator signal to a level suitable for injection into the system, after passing through a high-voltage coupling capacitor and an isolation transformer. This stage is implemented using a high-power operational amplifier supplied by ±15 V rails, allowing an output swing of up to approximately 30 Vpp under appropriate loading conditions. It should be noted that the choice of a power operational amplifier, rather than an instrumentation amplifier, is driven by the required output current levels. Instrumentation amplifiers are typically optimized for precision and high input impedance, but are not designed to supply the current levels demanded by the application. In contrast, the selected amplifier topology provides sufficient voltage and current capability to ensure proper signal injection into low-impedance fault paths. The schematic is presented in Figure 4.

**Coupling**. This module consists of a transformer responsible for increasing the alternating voltage so that it can emulate actual leaks in real situations, in addition to an output circuit that limits the maximum current and isolates DC voltages from the circuit.

Considering the architecture proposed, the device main specifications can be summarized as:

- It generates a sinusoidal signal to be detected by the technical team in case of a leakage/insulation failure;
- Controllable variable frequency: ranging from 30 to 80 Hz;
- Controllable variable voltage: ranging from 0 to 60 V—set after the signal generation, at the amplification stage;
- Maximum output test current: 60 mA;
- Voltage and frequency adjustment via potentiometer, allowing for a wide range of test currents;
- Power supply via grid connection or batteries.

The main feature of the proposed design is the ability to deliver test currents up to approximately 60 mA. However, it is important to clarify that no active current regulation mechanism is implemented. Instead, the current is passively limited by the maximum output voltage of the circuit in combination with a series output resistor in the coupling stage. Under short-circuit conditions, this configuration restricts the output current to approximately 60 mA, while for higher impedance fault conditions, the current decreases according to the load. Therefore, the delivered current is inherently dependent on the impedance of the fault path.

The output voltage is adjusted between the signal generation and amplification stages using potentiometers, which indirectly controls the resulting current level. The output impedance is predominantly defined by the series resistor at the coupling stage, which ensures safe operation by preventing excessive current. Additionally, the circuit allows frequency variation up to 80 Hz, which is advantageous for distinguishing the injected signal from typical 60 Hz grid interference.

### 3.2. Advantages of Its Implementation

Common commercial methods often rely on the principle of using a self-adaptive current (passive action), which depends on the power supply of the circuit being investigated and on the overall impedance of the faulty circuit [16]. As explained in the previous section, the proposed device allows manual control of both frequency and output voltage, enabling the adjustment of the injected current over a wide range according to the circuit requirements. This control is performed directly by the operator through potentiometers, and once set, the operating conditions remain fixed during the test. It is important to distinguish this approach from adaptive control strategies, such as those employed in Bender insulation monitoring devices, where the injection parameters are automatically adjusted in real time. In the proposed system, no automatic adaptation is implemented; instead, the flexibility lies in the operator-defined settings, which can be configured prior to the measurement according to the specific test scenario.

A major advantage, also seen in other commercial devices, is the ability to use the device in a predictive application without needing to shut down the system. This feature allows identifying traces of failure while the operation is kept unrestricted, resulting in higher availability for the installation and reducing risks related to unscheduled breaks.

A second advantage that also impacts the ability to work in an energized system is the possibility of injecting a controlled current signal, which is alternating—in contrast to the direct current that runs through the system, whose flow does not interfere with operation, resulting in fewer system interruptions and less operational risk. In fact, this controlled current, which is configured to vary until 60 mA, provides a wide range of reach to the tests, allowing faults to be found quite far from the injection source and also in a shorter time (compared to the main commercial alternative, the Bender device).

In addition, the frequency adjustment is also a distinguishing feature of the proposed device, which allows, through the potentiometer installed in the oscillator stage, adjusting the test so that a signal of a specific frequency is injected and detected accurately, without suffering interference from noise.

Finally, another characteristic already mentioned is the addition of an internal independent power supply, capable of powering the device in situations where connection to the electrical grid is not possible. This can be useful in remote areas, providing mobility to the device and increasing its flexibility of use.

For clarity, the main characteristics of the proposed signal injector can be summarized as follows:

- Adjustable output current range from 0 up to approximately 60 mA, defined by manual control of the output voltage;
- Passive current limiting behavior, with a maximum current of approximately 60 mA under short-circuit conditions;
- Output current dependent on the fault impedance, due to the absence of active current regulation;
- Electrical isolation from the DC bus through a coupling capacitor, ensuring no direct DC interaction with the monitored system;
- Capability to inject an AC test signal with configurable frequency, improving noise discrimination during fault detection.

## 4. Tests and Experimental Results

**Figure 5:**
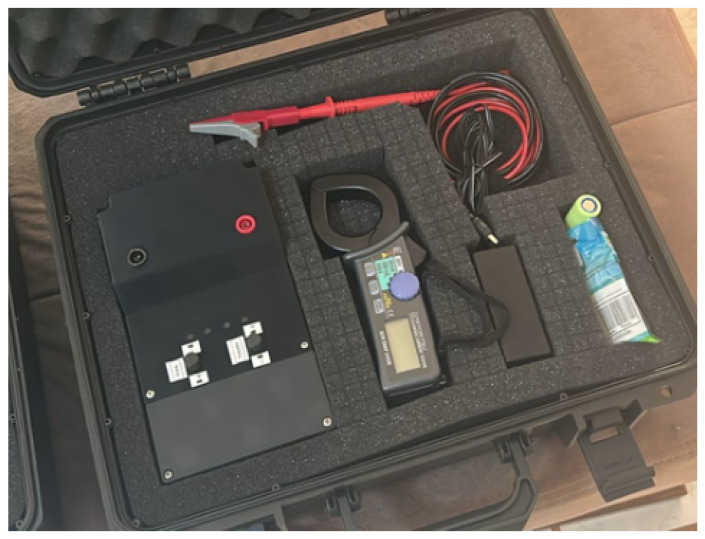
Kit with the leakage current device.

The performance and validation of the presented leakage current device are verified through tests and analyses in an operational environment just like the one where the first idea of development was conceived. For proper implementation, the signal injector is used along with a commercial clamp, being depicted as a kit containing all the apparatus needed in Figure 5.

**Figure 6:**
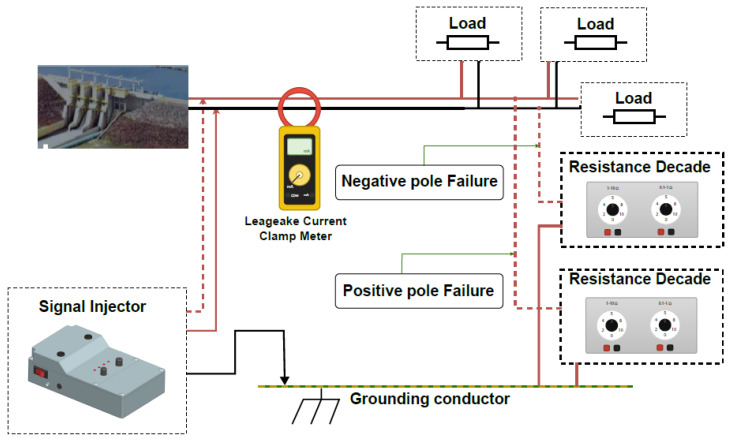
Operational diagram for using the leakage current device. Red and black lines represents positive and negative conductors, respectively, of the DCAS system and leakage. Red dashed lines represents the connection of the decade boxes to simulate the failures.

It is worth noting that most of the tests here aim to prove the advantages of using the proposed alternative device against a widely known and accepted commercial device. Figure 6 shows an example of its use, with two decade boxes being used to simulate failures in the negative and positive poles.

The experiments conducted in a real-world situation are presented and discussed in the sequence. During all experimental procedures, the connection and disconnection of the device were performed under energized conditions. No observable voltage disturbances, relay misoperations, or instability in the DC bus were reported, indicating that the interaction between the device and the system remained within acceptable operational limits.

### 4.1. Test Case 1—Local DC System with Controlled Leakage Simulation

#### 4.1.1. Description of Test Case 1

**Figure 7:**
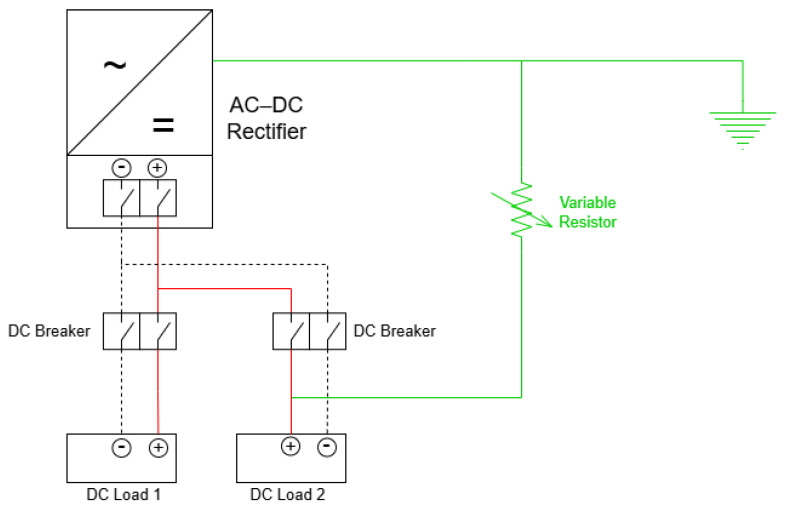
Schematic diagram of Test Case 1. Red line and black dashed line represents positive and negative conductors of the DCAS system, respectively.

The first test case was conducted on a local auxiliary DC system supplied by a 125 V AC–DC rectifier, representative of typical auxiliary system applications in hydroelectric power plants. This system supplies two local DC loads (two protection relays) through two dedicated circuit breakers and operates under an isolated (ungrounded) DC configuration. Figure 7 presents the schematic diagram of this test case. Ground leakage simulation was performed by connecting a variable resistor between one of the DC poles (positive or negative) and the protective earth (PE) conductor. This approach enabled the controlled reproduction of different fault levels, such as insulation degradation, ranging from high-resistance conditions (initial or incipient fault states) to low-resistance faults.

Both the proposed leakage current detection device and the commercial Bender equipment were independently connected to the DC bus at the rectifier panel, allowing a direct comparison between the two detection strategies. The signal injection point was located upstream of the feeder circuit breakers, ensuring that the injected signal propagated throughout the entire local DC system.

The resulting leakage currents were measured using a commercial clamp ammeter, positioned on the DC conductors and on the grounding conductor, as applicable. This test case was primarily employed to evaluate the operating principle of the proposed device, to verify its operation with signal injection applied to only one DC pole, and to compare the behavior of active current injection with the self-adaptive injection strategy implemented in the commercial equipment.

**Figure 8:**
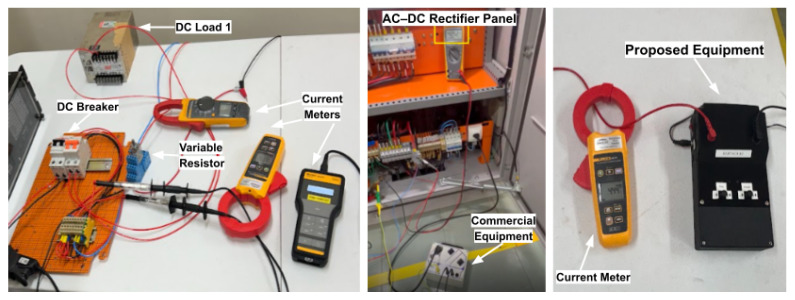
Test setup for Case 1, highlighting the main components employed in the execution of the experiments.

Figure 8 presents the test setup for Case 1, highlighting the main components employed in the execution of the experiment and in the acquisition of measurement data. The measurement system includes a Fluke 190-204 ScopeMeter (4 channels, 200 MHz bandwidth, 5 GS/s sampling rate, with an accuracy of ±1.5%, Fluke manufacturer, United States) and a Fluke 369 FC Leakage Current Clamp (current measurement accuracy of ±1.0%). Both instruments were used to observe low-level currents and waveform characteristics, ensuring adequate measurement resolution and signal-to-noise ratio for the proposed methodology.

#### 4.1.2. Results and Comparative Analysis for Test Case 1

**Figure 9:**
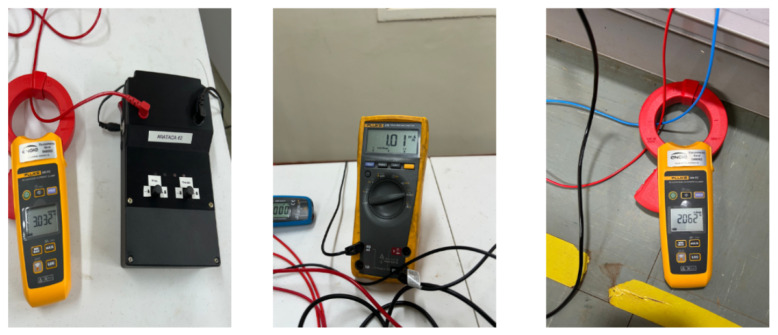
Measurements acquired using commercial clamp ammeter and multimeter: (**left**) injected current, (**center**) leakage current, and (**right**) current in the rectifier branch.

In the local DC system configuration, the tests were carried out with the rectifier supplying two protection relays, allowing the controlled simulation of a ground leakage fault through an adjustable resistor directly connected to one of the relays (DC Load 2). The main objective of this test was to evaluate the capability of the proposed device to detect and locate high-impedance leakage faults, as well as to compare its performance with that of a widely used commercial device. Table 1 presents the results obtained for a leakage resistance of 9 kΩ, with the injected signal frequency maintained at 60 Hz while the injection current of the proposed device was incrementally varied at the negative pole. Figure 9 shows a record of the measurements performed during the ground leakage test with a 9 kΩ resistance and an injected current of 3 mA, including the measured values of the injected current, the leakage current, and the current flowing through the rectifier branch.

The results demonstrate an approximately linear relationship between the injection current configured in the proposed device and the currents effectively measured both in the rectifier branch and along the leakage path. A linear regression analysis was performed, yielding a strong correlation between the variables ($R^{2}\approx 0.99$), confirming the proportional behavior observed experimentally. This relationship indicates that the injected current is distributed in a predictable manner among the circuit branches, according to the associated impedances, enabling selective identification of the faulty branch even under low absolute current conditions. This behavior highlights the active control of the test current implemented in the proposed device, resulting in repeatable and traceable measurements. Such characteristics are particularly relevant for diagnostic applications, where quantitative interpretation of the results is essential for accurate fault localization.

For comparison purposes, Table 2 presents the results obtained with the commercial Bender equipment under equivalent test conditions. The commercial system used was the Bender EDS195P (Bender manufacturer, Germany) with PSA clamp (PSA3020/PSA3052), which injects test signals in alternating pulse cycles (approximately 6 seconds). The first test was performed under a high-impedance ground leakage condition using the same 9 kΩ resistance applied in the tests with the proposed device. In this scenario, the injected current was very low (below 5 mA), such that the currents in the rectifier branch and at the leakage point were not registered by the Bender equipment. Consequently, the same topology was tested using a lower ground leakage resistance (200 Ω). When the leakage was emulated on the negative pole, the injected current reached 8 mA, allowing measurement in the rectifier branch (6 mA), but not directly in the leakage branch. By performing a direct measurement on the protective earth (PE) conductor of the rectifier circuit, ground leakage detection was achieved with the Bender equipment. However, when the same test was conducted on the positive terminal, the adaptive injected current remained below 5 mA, preventing ground leakage detection with the commercial device.

In the tests performed with the commercial equipment, it was observed that fault detection depends on measuring the integral current flowing through the protective earth (PE) conductor of the device itself, using a dedicated current clamp (PSA3020) associated with the Bender EDS195P system. Currents below approximately 2 mA, which corresponds to the practical sensitivity limit of the equipment, were not consistently captured, even when the leakage was effectively present and detectable through direct current measurements using commercial clamp ammeters.

Furthermore, the self-adaptive injection method employed by the commercial equipment, based on periodic alternating pulse cycles (with a period on the order of 6 seconds), resulted in injected currents whose magnitude did not exhibit a direct proportional relationship with the applied leakage impedance. This behavior hindered the quantitative interpretation of the results, which is essential for reliable measurement analysis and accurate fault localization.

### 4.2. Test Case 2—Distributed DC System with Remote Protection and Control Panel

#### 4.2.1. Description of Test Case 2

**Figure 10:**
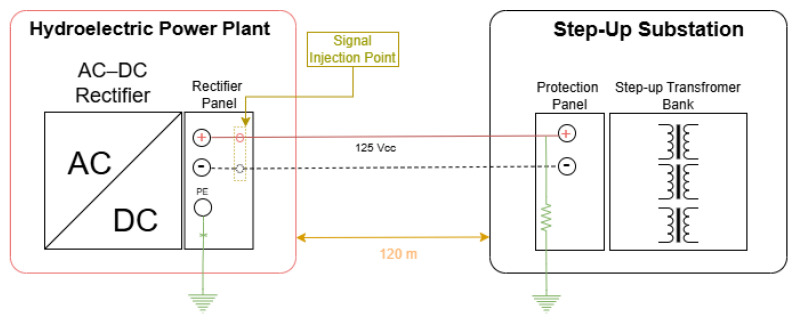
Schematic diagram of Test Case 2. Red line and black dashed line represents positive and negative conductors of the DCAS system, respectively.

The second test case reproduces a more complex topology that is representative of real-world applications in hydroelectric power plants, involving a distributed DC system between the powerhouse and the step-up substation. In this configuration, the AC–DC rectifier and its control panel are located in the powerhouse of the hydroelectric plant, while the protection and control panels associated with the step-up transformer bank are installed at the substation, at an approximate distance of 120 m. The 125 V DC bus supplies the remote panels through long cable runs. Ground leakage simulation was performed at the remote protection panel by connecting power resistors between one of the DC poles and the local grounding system of the substation. Figure 10 presents the schematic diagram of the tests conducted under Test Case 2.

For both the proposed leakage current detection device and the commercial Bender equipment, the signal injection point was located at the rectifier panel in the powerhouse. The injected signal propagated along the DC conductors to the remote panel at the substation, closed the circuit through the ground leakage path via the test resistor, and returned through the grounding system (protective earth, PE, conductor of the rectifier), enabling the evaluation of the global circulation of the test current in an extensive DC system.

**Figure 11:**
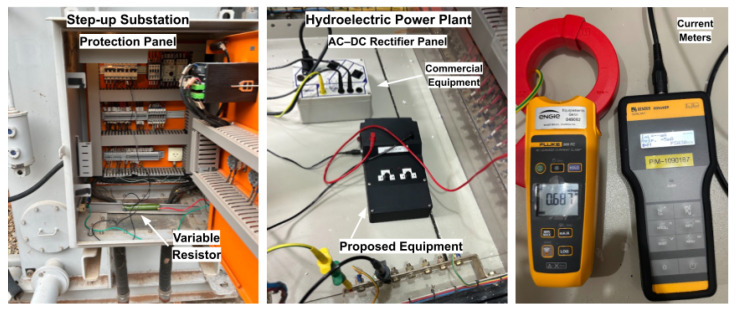
Test setup for Case 2, highlighting the main components employed in the execution of the experiments.

Current measurements were performed at different points of the system, including the rectifier panel, the interconnecting conductors, and the protection panel at the substation. This test case enabled the assessment of leakage detection capability over long distances, the comparison of the active injection method with the behavior of the commercial equipment under higher system impedance conditions, and the demonstration of the proposed device operation without the need for system de-energization or load disconnection. Figure 11 presents the test setup for Case 2, highlighting the main components employed in the execution of the experiment and in the acquisition of measurement data.

#### 4.2.2. Results and Comparative Analysis for Test Case 2

The tests performed with the proposed device were conducted for different leakage resistance values (639 Ω, 200 Ω, and 39 Ω) connected to the positive pole, initially maintaining the injection frequency at 60 Hz and subsequently adjusting it to 40 Hz in order to investigate the influence of frequency on the circulation and detectability of the test current. Table 3 summarizes the results obtained with the proposed device.

The results indicate that the proposed device maintained its capability to inject and detect leakage current even under this extended DC system configuration. It is observed that, for a leakage resistance of 39 Ω, reducing the injection frequency from 60 Hz to 40 Hz resulted in a significant increase in the current measured at the leakage point, from approximately 8.15 mA to 17.9 mA. This behavior experimentally confirms that adjustment of the injection frequency is a determining factor for mitigating the effects of distributed cable capacitance and for maximizing the signal-to-noise ratio at the measurement point. In addition, the current distribution between the rectifier branch and the leakage path remained consistent with the associated impedances, allowing clear identification of the fault location even under increased signal attenuation conditions when compared to Test Case 1.

For comparison purposes, Table 4 presents the results obtained with the commercial Bender equipment under the same test conditions.

In the tests conducted with the commercial equipment, it was observed that the current effectively detected in the distributed DC system remained close to the lower sensitivity limit of the instrument, regardless of the applied leakage resistance value. Under these test conditions, the built-in meter of the equipment was unable to assign a numerical value to the measured current, even when measurements were performed directly on the protective earth (PE) conductor. Consequently, a conventional clamp ammeter was required to confirm the presence of current at the leakage point. The values obtained with the conventional instrument revealed leakage current amplitudes below the sensitivity threshold of the Bender equipment for the adaptive injection current levels generated by the device.

Additionally, a test was performed using a leakage resistance of 39 Ω with the PE conductor of the rectifier panel disconnected, which is a procedure recommended in the operating manual of the Bender equipment. In this configuration, the Bender instrument was able to detect the presence of leakage current at the fault location; however, it was not able to assign a numerical value to the measurement due to the low current amplitude.

To provide a theoretical interpretation of the observed frequency-dependent behavior, the distributed DC system can be approximated by a simplified equivalent circuit, in which the leakage path is represented by a resistance in parallel with an equivalent capacitance to ground. This capacitance accounts for the distributed parasitic capacitances of the DC cables along their length. In this representation, the impedance of the capacitive path is frequency-dependent and can be expressed as: $X_{c}=1/2\pi fC$.

As the frequency increases, the capacitive reactance decreases, allowing a larger portion of the injected current to flow through the capacitive path instead of the leakage resistance. Conversely, at lower frequencies, the capacitive reactance increases, reducing the current diverted through the capacitive path and consequently increasing the current flowing through the leakage resistance.

This behavior is consistent with the experimental results obtained in Test Case 2, where reducing the injection frequency from 60 Hz to 40 Hz resulted in a significant increase in the measured leakage current at the fault location. Therefore, the frequency adjustment capability of the proposed device plays a key role in mitigating the effects of distributed capacitance and improving fault detectability in extended DC systems.

### 4.3. Discussion

The experimental results presented herein allow a comprehensive assessment of the performance of the proposed device under different application conditions, encompassing both a local DC system and a distributed DC system with long cable runs. The combined analysis of the two test cases highlights fundamental differences between the active current injection strategy adopted by the proposed device and the self-adaptive injection method employed by the commercial device used as a reference.

In Test Case 1, corresponding to a local DC system with controlled simulation of ground leakage, the results demonstrated that the proposed device was able to consistently detect and locate high-impedance faults (9 kΩ). The approximately linear relationship observed between the configured injection current and the currents measured at the rectifier branch and along the leakage path indicates that the active injection method provides predictable and repeatable measurements. This behavior is particularly relevant for diagnostic applications, as it enables not only fault detection but also a quantitative interpretation of current distribution within the system, thereby supporting selective identification of the faulty branch. In contrast, the tests performed with the commercial device revealed limitations in the detection of high-impedance leakage faults. The current injected by the self-adaptive method exhibited low magnitude and weak correlation with the applied leakage impedance, resulting in difficulties in both measurement and result interpretation. Furthermore, the reliance on measuring the integral current flowing through the protective earth (PE) conductor to achieve successful detection limits the fault localization capability in test conditions where the leakage current magnitude is low.

In Test Case 2, which reproduces a distributed DC system with approximately 120 m of cabling between the rectifier and the remote protection and control panels of the step-up transformers, the differences between the two approaches became even more pronounced. The proposed device maintained the ability to inject and detect leakage currents under all tested configurations, including relatively high leakage resistances, demonstrating robustness against the effects of distributed cable capacitances, which attenuate the injected AC signal. The results obtained from varying the injection frequency experimentally confirmed that this parameter has a significant influence on the circulation of the test current, enabling optimization of the signal-to-noise ratio and improved fault detectability in extensive DC systems. Conversely, the commercial device exhibited limited performance in this test case. The effectively detected current remained close to the lower sensitivity threshold of the instrument, regardless of the applied leakage resistance, requiring additional procedures, such as disconnecting the PE conductor, to enable detection. Although such procedures are described in the manufacturer’s documentation, they represent an important operational limitation, as they require modifications to the system topology during the diagnostic process.

It should be noted that the leakage conditions in the experimental setup were emulated using fixed resistive elements, which provide a simplified representation of real faults. In practical applications, leakage paths may present non-linear and intermittent characteristics due to environmental and insulation conditions. Although this simplified approach enables controlled and repeatable testing, further investigations considering dynamic and non-linear leakage behaviors are recommended for future work.

Overall, the results indicate that the active injection strategy, with independent control of current and frequency, offers significant advantages in terms of sensitivity, operational flexibility, and clarity in the interpretation of measurements.

## 5. Conclusions

This paper presented the development and experimental validation of an alternative device for the detection and localization of leakage currents in auxiliary direct current systems (DCAS), with a focus on typical applications in hydroelectric power plants and substations. The performance of the device was evaluated in a real operational environment through comparative tests against a commercial device widely used in the electrical power sector. The experimental results demonstrated that the proposed device is capable of detecting and locating ground leakage faults in energized DC systems, both in local configurations and in distributed systems with long cable runs. The active and controlled current injection strategy enabled reliable identification of high-impedance faults, overcoming limitations observed in the self-adaptive injection method employed by the commercial device, particularly under low leakage current conditions and in extensive DC systems. The main advantages observed for the proposed device include: (i) independent control of the test signal current and frequency, allowing greater predictability and adaptability to the electrical characteristics of the system under evaluation; (ii) the ability to operate with signal injection applied to only one pole of the DC system; (iii) suitability for application in distributed systems without the need for system de-energization or grounding reconfiguration; and (iv) structural simplicity and low implementation cost, which favor large-scale adoption in maintenance, commissioning, and diagnostic activities. Overall, the results confirm the technical feasibility of the proposed device as an effective and low-cost alternative for the detection and localization of leakage currents in auxiliary DC systems.

## Figures and Tables

**Table 1:** Test results obtained with the proposed leakage current detection device in Test Case 1.

Impedance	Injected Current	Injected Voltage	Measured at the Rectifier	Measured at the Leakage Point	Successful Detection
9 kΩ	3 mA	9.0 Vac	2.0 mA	1.0 mA	Yes
9 kΩ	5 mA	15.0 Vac	3.5 mA	1.6 mA	Yes
9 kΩ	10 mA	31.7 Vac	6.8 mA	3.4 mA	Yes
9 kΩ	15 mA	47.0 Vac	10.0 mA	5.0 mA	Yes

**Table 2:** Results of the test performed with the commercial Bender equipment in Test Case 1.

Pole	Impedance	Injected Current	Injected Voltage	Measured at the Rectifier	Measured at the Leakage Point	Successful Detection
Negative	9 kΩ	<5 mA	20 Vdc	Not measured ^1^	Not measured	No
Negative	200 Ω	8 mA	20 Vdc	6 mA	Detected current ^2^	Yes
Positive	200 Ω	<5 mA	20 Vdc	Not measured	Not measured	No

^1^ The Bender equipment failed to measure the current due to insufficient signal amplitude. ^2^ Detected current, but not quantified (below sensitivity limit).

**Table 3:** Experimental results of the proposed device in Test Case 2 (distributed DC system with a remote protection and control panel).

Impedance	Injected Current	Injected Voltage	Signal Frequency	Measured at the Rectifier	Measured at the Leakage Point	Successful Detection
639 Ω	13.0 mA	14.2 Vac	60 Hz	8.6 mA	2.8 mA	Yes
200 kΩ	9.3 mA	10.0 Vac	60 Hz	6.5 mA	4.7 mA	Yes
39 kΩ	10.0 mA	10.0 Vac	60 Hz	1.9 mA	8.2 mA	Yes
39 kΩ	22.0 mA	23.0 Vac	40 Hz	3.9 mA	17.9 mA	Yes

**Table 4:** Experimental results of the commercial Bender device in Test Case 2 (distributed DC system with a remote protection and control panel).

Impedance	Injected Current	Leakage Current (Bender)	Leakage Current (Commercial Meter)	Successful Detection?
639 Ω	<25 mA	Not measured ^1^	0.687 mA	No
200 Ω	<10 mA	Not measured ^1^	0.687 mA	No
39 Ω	<10 mA	Not measured ^1^	0.687 mA	No

^1^ The Bender equipment failed to measure the current due to insufficient signal amplitude.

## Data Availability

The original contributions presented in the study are included in the article, further inquiries can be directed to the corresponding author.

## Abbreviations

The following abbreviations are used in this manuscript:

DCAS	Direct Current Auxiliary Systems
FDR	Frequency Domain Reflectometry
IMD	Insulation Monitoring Devices
TDR	Time Domain Reflectometry
AC	Alternating Current
DC	Direct Current
PE	Protective Earth
